# In-Plane Seismic Strengthening of Brick Masonry Using Steel and Plastic Meshes

**DOI:** 10.3390/ma15114013

**Published:** 2022-06-06

**Authors:** Safi Ullah, Syed Hassan Farooq, Muhammad Usman, Burhan Ullah, Manzoor Hussain, Asad Hanif

**Affiliations:** 1 Military College of Engineering (MCE), National University of Sciences and Technology (NUST), Risalpur 24090, Pakistan; safiuet1@gmail.com (S.U.); syed2arqam@gmail.com (S.H.F.); burhanullah4477@gmail.com (B.U.); manzoor7@yahoo.com (M.H.); 2 School of Civil and Environmental Engineering (SCEE), National University of Sciences and Technology (NUST), Sector H-12, Islamabad 44000, Pakistan; 3 Civil and Environmental Engineering Department, King Fahd University of Petroleum and Minerals (KFUPM), Dhahran 31261, Saudi Arabia; 4 Interdisciplinary Research Center for Construction and Building Materials, King Fahd University of Petroleum and Minerals (KFUPM), Dhahran 31261, Saudi Arabia

**Keywords:** brick masonry, seismic behavior, strengthening, mechanical performance

## Abstract

Unreinforced masonry structures are vulnerable to seismic loading due to their brittle behavior, and must therefore be strengthened. This paper presents the seismic performance of brick masonry strengthened with steel and plastic meshes. For this purpose, twenty masonry wallets of (600 × 600 × 113 mm) were constructed, keeping the same materials and workmanship. Fifteen of them were reinforced using steel and plastic meshes. These specimens were tested for in-plane static cyclic diagonal tension (shear) behavior. The critical parameters, such as shear stress, strain, failure modes, ductility, energy dissipation, and stiffness degradation were investigated. Compared to reference and plastic-reinforced specimens, the steel-reinforced samples were found to be highly effective. Furthermore, the recommended category of steel increased the shear capacity, energy dissipation, and ductility ratio by 1.3, 14, and 6.3 times, respectively.

## 1. Introduction

Brick masonry is the oldest and most widely used form of construction [1,2,3]. It is preferred because of its availability, high compressive strength, heat absorption, good soundproofing, and low cost. Although it is the oldest construction method, masonry construction is not well understood in terms of its heterogeneity and anisotropic behavior. Furthermore, compared to other materials such as concrete and steel, it is challenging to analyze, design, and explain the behavior of masonry [4]. Its properties depend upon the ingredients’ manufacturing process and differences in the constituent materials. Typical brick masonry structures are made in box-shaped structures where the walls are load-bearing. These structures are often strengthened for out-of-plane stresses via different methods such as ring beams and concrete columns. These measures confine masonry and strengthen it for the in-plane as well [5]. However, the walls are mostly pierced for windows and doors. Therefore, stresses can accumulate at the corners of these walls.

In Pakistan, almost all old residential buildings and architectural heritage buildings are constructed of brick masonry. These structures are prone to earthquakes, especially in the northern and northwestern regions of the country. Because Pakistan is located on the Indian plate beside the junction between the Eurasian and Indian plates, these plates have been slowly converging for millions of years, producing many faults. The most critical faults include the Main Karakorum thrust, Main mantle thrust, Main boundary thrust, Margalla, and Chaman faults [6].

The brittle behavior and low tensile strength of masonry can be hazardous to both property and human lives. As observed in the earthquake of 8 October 2005 in Pakistan, around 74,000 were killed, 70,000 injured, and 2.5 million families displaced. Most of the damage was observed in Kashmir, where traditional brick masonry structures are widely used. Indeed, the same construction practices are followed all over the country [7]. Therefore, the demand for strengthening of these vulnerable structures increases day by day [6,8]. Many existing structures in Pakistan are built without following any national building code for masonry, as no such national provision exists for masonry structures [9].

Unreinforced brick masonry is primarily designed for gravity load by utilizing its compressive strength capacity. It is a non-tensile material, as it barely takes any load in tension [10]. Under compressive load on masonry, mortar attempts to expand due to its Poisson ratio. The bond between mortar and brick confines the mortar’s deformation, producing lateral tensile stresses in bricks and tri-axial compressive stresses in mortar. The mortar crushed under pressure forms a weak zone, and the lateral tension between surface particles of the brick initiates cracks that propagate from the micro-level to the macro-level [11].

During a seismic event, masonry building structures are under flexural loading. Masonry walls are subjected to out-of-plane flexural stresses and in-plane shear stresses, especially diagonal compression. The maximum stresses are located at the toe, the center of the wall, the heel, and the point of load application. The non-homogeneous and anisotropic behavior of masonry makes it very difficult to predict the performance of brick masonry under such loading conditions. Thus, the design of such a structure requires maximum awareness, and therefore, it should be reinforced.

In the last few decades, many strengthening techniques have been proposed to overcome the vulnerability of masonry structures. Several strengthening methods and materials proposed by various researchers are glass, carbon fiber-reinforced polymers, flax fibers, ferrocement, FRP, surface mounted, and galvanized steel strips to resist corrosion [12,13,14,15,16,17,18,19,20]. Furthermore, numerical modeling techniques developed by researchers [21] can be used to predict the life of such steel reinforcements. Recently Angiolilli et al. [14] have used Glass FRCM with a lime-based mortar to retrofit ancient masonry structures. The mortar matrix initially carries the load; when multitracking begins, the load is partially transferred to the FRCM, and by the end, the total load is carried by the FRCM. According to the author, this method is more compatible with the intrinsic properties of ancient masonry. In other research, Giuseppe Ferrara et al. [16] used a textile-reinforced mortar (TRM) on the surface of masonry and tested it in diagonal tension. This method increases the strength and ductility of the masonry; however, it has limitations in terms of its post-elastic behavior. The post-peak behavior can be further improved using ductile and sustainable filler material, as has been proposed by Foti et al. [22]. Following the same diagonal tension test method, Yavuz Yardim et al. [23] used polypropylene fiber-reinforced mortar plastering and ferrocement. The polypropylene and ferrocement exhibit a significant increase in the shear strength of the masonry.

Similarly, Francesca Ferretti et al. [24] have investigated the use of an FRCM Basalt grid, Aramid glass grid, and strips of steel-reinforced grout (SRG). The grids are anchored using steel rods in pre-drilled through-holes. This technique plays a crucial role in enhancing the shear strength capacity. Luis Mercedes [25] used hemp, vegetal meshes, and glass fibers as strengthening materials. The materials were embedded between two layers of plaster. Although the vegetal mesh presented a debonding problem, this material showed superior efficiency in enhancing the shear strength. Sabbà et al. [26] used straw fibers to increase the compressive and flexural strength of earthen mortar. Behera et al. [27] used a geogrid-reinforced mortar in the bed joints and bed-head joints of the masonry. While this increases the shear strength and ductility of the masonry, applying this material to every joint is challenging. Deng-Hu Jing et al. [28] proposed a different technique, in which they used pre-stressed steel strips on masonry walls vertically and horizontally. This method is efficient for enhancing lateral load capacity and crack initiation behavior; however, due to the complexity of masonry, a complicated calculation is involved in determining stress losses due to strip relaxation and creeping in the masonry.

While these proposed methods are strength efficient, they have associated compatibility, cost, or materials installation issues. Therefore, there is a need to research strengthening techniques based on optimizing their cost, strength, and installation efforts.

In this research, we endeavored to find the optimum materials for the in-plane shear strength of brick masonry. The primary test performed here was the diagonal tension test under cyclic loading, for which surface-mounted steel and plastic polymer mesh were used. Anchoring the surface reinforcement is a challenge, and therefore efforts were made to develop a simplified technique. A diagonal tension test under cyclic loading was carried out along with prism and mortar compression tests in order to experimentally analyze the effectiveness of the methods used. This test method is widely used for analyzing shear capacity and other essential parameters. Previous authors have proposed multiple approaches to finding the shear, tensile strength, and other parameters of masonry using the diagonal tension test [10,29,30]. The ASTM E-519 provides an approach to calculating the shear capacity of masonry panels under diagonal tension loads, considering that the tension at the center of the panel is in pure shear and its main direction passes through the diagonal corners.

### Research Aims and Objective

The aim of this work is to develop a new technique for the strengthening of the existing brick masonry structures. The primary objective is to evaluate the in-plane seismic shear performance of steel and plastic-reinforced masonry wallets and the influence of mesh configuration.

## 2. Materials and Experimental Program

This research includes the assessment of steel and plastic-strengthened clay brick masonry wallets and a comparison with unreinforced wallets. The material characteristics were tested initially, then the compressive strength of the prisms was tested; finally, the wallets were tested for shear strength.

### 2.1. Materials Characteristics

**Figure 1:**
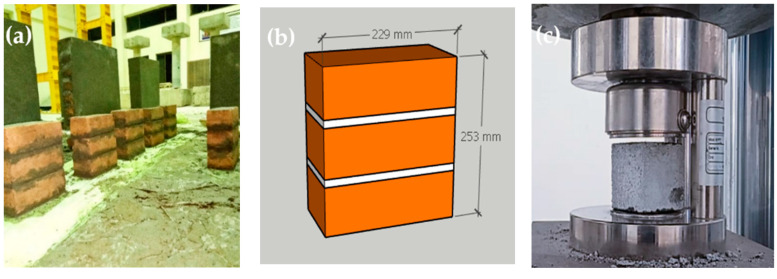
Masonry prism and mortar cubes. (**a**) Prism specimens during curing; (**b**) Prism dimensions; (**c**) Mortar cube compression.

In this research, locally and commercially available industrial clay bricks were used. The brick dimensions ere 114.3 × 76.2 × 229 mm. For compressive strength, six prism triples with an aspect ratio of 2.21, as shown in Figure 1a,b, were tested as per ASTM C-1314. The average compressive strength of the brick prisms was 5.57 MPa, with a standard deviation of 0.94 MPa. N-type mortar with 1:4 (cement: sand) was used in the masonry wallets, and 50 mm Mortar cubes, as shown in Figure 1c, were tested for compression under ASTM-C109 [31]. The average compressive strength of the mortar cubes was 15.6 MPa, with a standard deviation of 0.83 MPa.

### 2.2. Sample Preparation

**Figure 2:**
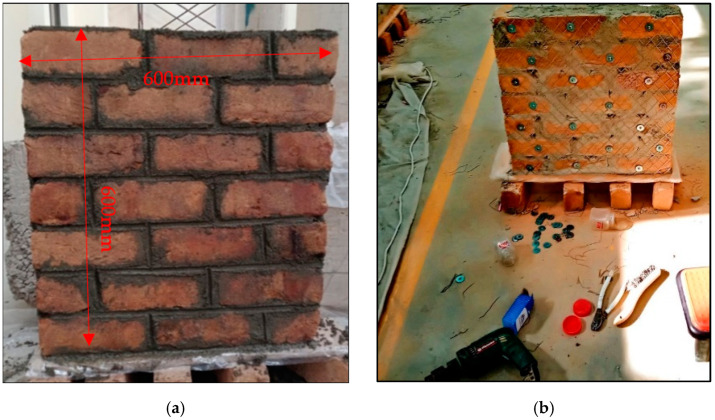
Preparation of samples. (**a**) Wallet dimensions; (**b**) Installation of reinforcement.

The principal aim of this research is to analyze the seismic performance of strengthened brick masonry. For this purpose, twenty masonry wallets of the same size (600 mm × 600 mm) were constructed in the laboratory according to ASTM E-519, as shown in Figure 2a, and cured for a minimum of 28 days. The same size of specimens has previously been used for testing by other researchers. Locally available materials (cement, sand, solid clay bricks) and artisans were used, as the intention was to represent the typical masonry of Pakistan. Single brick wallets of 113 mm were constructed. Five wallets were kept for comparison, and fifteen were strengthened using steel and plastic meshes. Table 1 shows the nomenclature of the strengthening scheme followed in this research.

**Figure 3:**
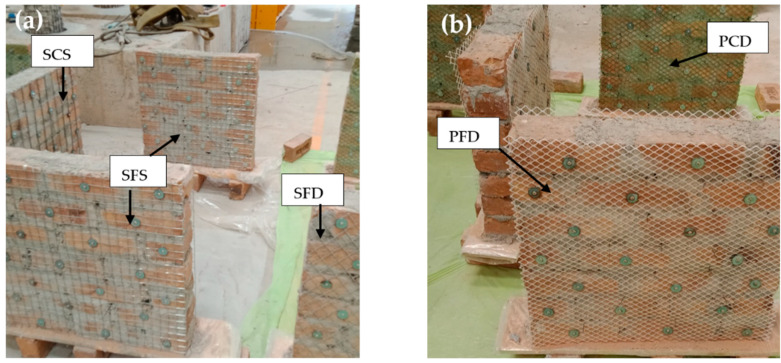
Pictural views of different meshing schemes. (**a**) Steel-reinforced specimens; (**b**) Plastic-reinforced specimens.

**Figure 4:**
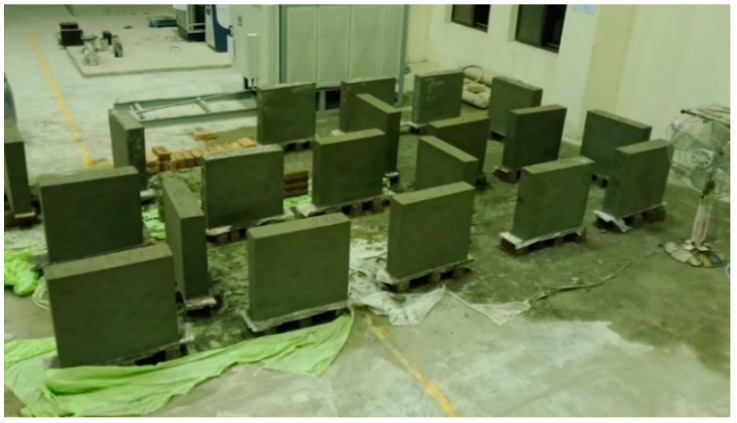
Masonry wallets after plastering.

As shown in Figure 3, The SFS specimen were reinforced with steel fine square mesh. Similarly, the SCS was reinforced with steel coarse square mesh. The quantity of reinforcement in the SFS and SCS specimens were almost the same, i.e., 0.73% and 0.74%, respectively, as listed in Table 1. The SFD specimens were strengthened using steel fine diagonal mesh, PCD specimens were reinforced with plastic coarse diagonal mesh, and PFD specimens were reinforced with plastic fine diagonal mesh. All specimens were strengthened symmetrically with the same vertical and horizontal reinforcement and along both front and back surfaces. The wallets were plastered with N-type mortar and then cured, as shown in Figure 4.

### 2.3. Steel/Plastic Strengthening

Steel and plastic mesh was used to strengthen the masonry wallets. The material properties and size details are provided in Table 1. Two varieties of plastic and three varieties of steel mesh were used. The mesh installation process was simple and easy. Initially, the points were marked using the mesh as a grid. Then, the wallets were drilled at the specified locations. As shown in Figure 2b, reinforcement meshes were anchored in every brick with the help of ¼10 pan head Philip steel screws and washers with a thickness of 1 mm and internal and external diameter of 6 mm and 40 mm, respectively.

### 2.4. Test Setup and Loading System

**Figure 5:**
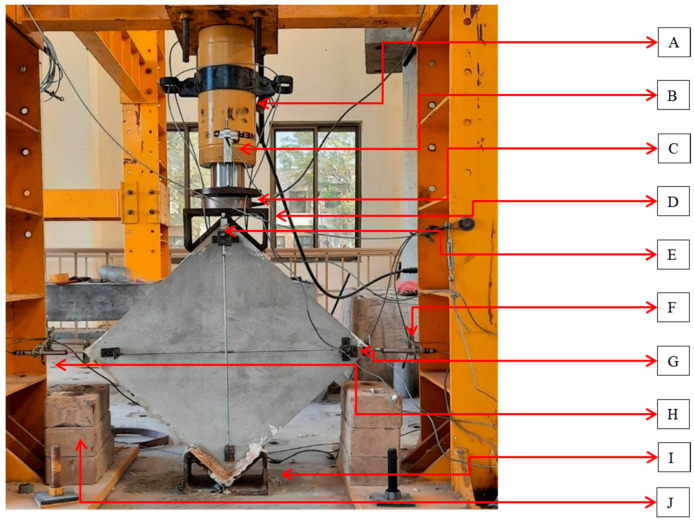
Diagonal tension testing instrumentation: (A) hydraulic jack; (B) top vertical LVDT; (C) 50-ton load cell; (D) top loading shoe; (E) internal vertical LVDT; (F) external horizontal right LVDT; (G) internal horizontal LVDT; (H) external horizontal left LVDT; (I) bottom loading shoe; (J) supports in case of sample collapse.

**Figure 6:**
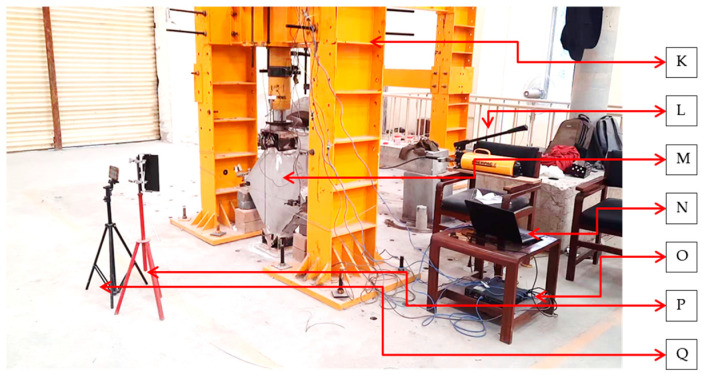
Diagonal tension (shear) testing setup: (K) shear frame (100-ton axial capacity); (L) hydraulic pump; (M) masonry wallet (test sample); (N) laptop for data reading and recording; (O) data logger; (P) lamp; (Q) video recording equipment.

**Figure 7:**
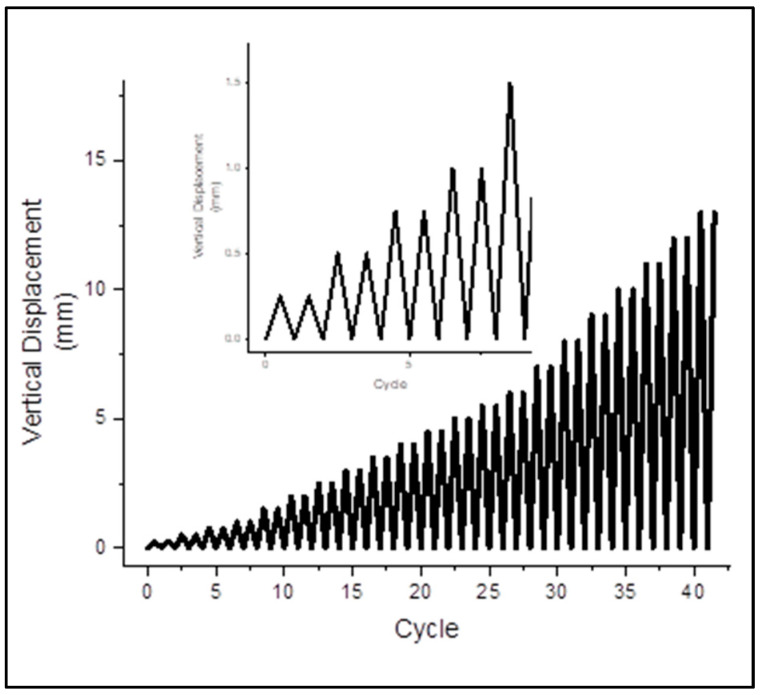
Loading history.

The test setup and loading system is shown in Figure 5 and Figure 6. The masonry wallets were tested in the Structure Dynamics Lab at the Military College of Engineering, Risalpur, Pakistan. Loading shoes were prepared as per the instructions in the ASTM-E519. Initially, the loading shoe was leveled on the ground, then filled with plastic-state gypsum. Prior to the initial setting of the gypsum, the wallets were placed and leveled. The same procedure was performed for both top and bottom loading shoes. Gypsum capping was provided in order to avoid local crushing of the wallet at the point of load application. The wallets with loading shoes were then manually dragged to the loading frame, aligned, and leveled to the hydraulic jack. The test assembly was prepared as per the ASTM-E519, with the load applied on the top shoe of the diagonally placed masonry wallet. This standard provides an approach to finding the shear stress from the diagonal compression using Equations (1)–(5), and ensures that the stresses along the compression diagonal are pure shear. In order to find the shear performance of the reinforced wallets under seismic conditions, cyclic loading was applied at the top loading shoe. Cyclic loading is reversal loading which degrades the structure with each cycle, and the damage is irreversible in the form of stored deformation. Such cyclic loading is applied in order to assess the seismic performance of structures. The data from the cyclic loading test can be used to find important seismic design-related parameters, such as energy dissipation, ductility, and stiffness degradation. [32]. The test was performed according to the cyclic loading protocols shown in Figure 7. The same (unidirectional) cyclic loading protocol has been previously used by Almeida et al. [33] and Dong et al. [34]. Up to 1 mm total vertical deformation, the loading increment was 0.25 mm. After a 1 mm vertical deformation, the increment becomes 0.5 mm. Then, after 5 mm of total vertical deformation, the increment is 1 mm. Each set of loading cycles was applied twice.

### 2.5. Instrumentation

The test assembly for the diagonal tension test on the wallets included a 100-ton capacity reversible hydraulic jack and five LVDTs with which to record deformations, as shown in Figure 5 and Figure 6. A Micro Measurement data logger was used to record deformations. A load cell of 50-ton capacity was used. Two strain gauges were installed on the front surface of the wallets, with a gauge length of 700 mm, as described in ASTM E-519. Externally, three strain gauges were installed, two on the horizontal corners and one on the top beside the hydraulic jack, in order to determine the vertical deformation of the jack loading head. For horizontal deformation, separate steel heads (V-shaped with a cap) were installed to measure the sway at the corners without dispositioning the LVDT plungers. Safety supports were provided to hold the wallets and to protect the instruments in case of collapse. Safety cables were wrapped around the frame and were not in contact with the wallets. The wires were provided to prevent the overturn of the specimen and ensure the safety of the installed instruments. No overturning was observed during the whole testing process. Figure 5 and Figure 6 show the test setup and strain gauge arrangement, respectively.

### 2.6. Test Observations

This section discusses the results, crack development, crack patterns, and capacities of the masonry wallets subjected to static cyclic diagonal tension testing. This test was performed in order to assess the contribution of reinforcement and compare it with unreinforced specimens. This assessment includes the failure behavior, load-carrying capacity, ductility, and energy dissipation.

#### 2.6.1. Reference Specimens

**Figure 8:**
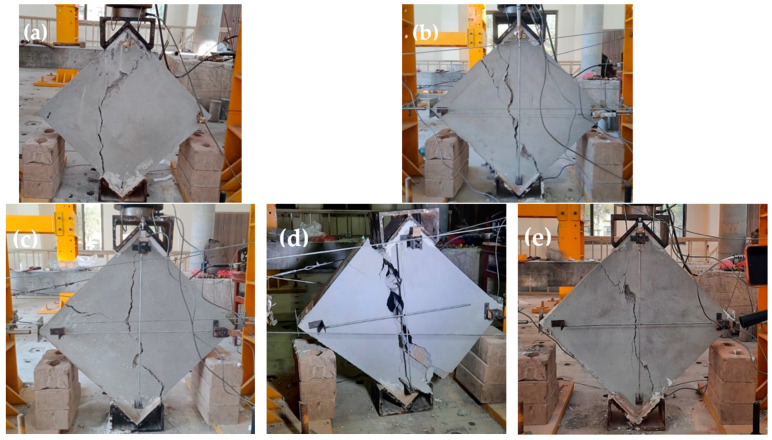
Crack patterns of REF specimens. (**a**) REF (1); (**b**) REF (2); (**c**) REF (3); (**d**) REF (4); (**e**) REF (5).

The REF specimens showed brittle shear failure with a sudden decrease in load-carrying capacity. A central shear crack split apart the wallets, as shown in the post-test images in Figure 8a–e. REF-4 wallets experienced total collapse, as shown in Figure 8d. The average vertical load taken by the unreinforced samples was 75 KN, with a shear stress of 0.78 MPa and shear strain of 0.087%. No localized failure was experienced. Stepped cracks were observed in three specimens, while diagonal shear with coarse slip was observed in the remaining two REF specimens. The mean stress achieved at the ultimate point (20% of the peak) was 0.62 MPa, corresponding to a strain of 0.36%.

#### 2.6.2. Plastic Reinforced Specimens

**Figure 9:**
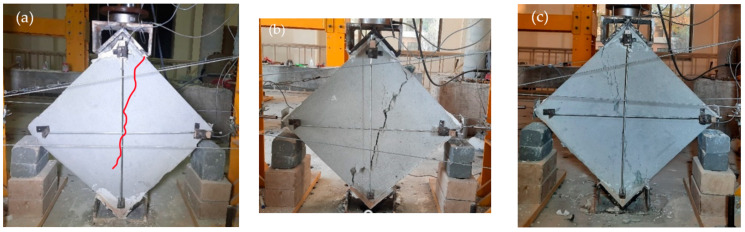
Failure patterns of PCD-reinforced wallets.

**Figure 10:**
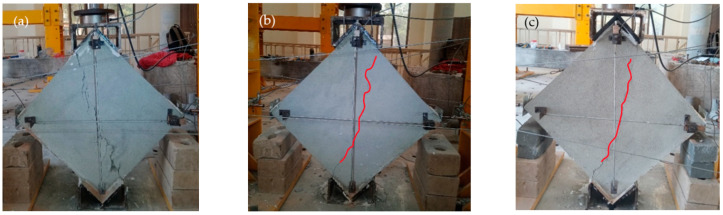
Failure patterns of PB reinforced wallets.

As discussed earlier, two configurations of plastic reinforcement were used, plastic coarse diagonal (PCD) and plastic fine diagonal (PFD). The PCD wallets showed less brittle behavior than the REF and PFD wallets. The PCD and PFD wallets were able to take a peak vertical load of 101.6 KN and 72.0 KN, respectively, and the peak stresses were 0.92 MPa and 0.96 MPa, corresponding to 0.14% and 0.093% strains. All plastic specimens experienced the same diagonal shear failure type, as shown in Figure 9a–c and Figure 10a–c. All plastic meshes yielded and were cut after excessive deformation only at the location of cracks, and were in un-disturbed condition at all other locations. The cracks were mildly brittle, and the specimens remained intact even after the end of the test. The ultimate stresses reached 0.73 MPa and 0.52 MPa, corresponding to 0.48% and 0.42% ultimate strains. The loading shoe provides confinement to the top and bottom portion of wallets; therefore, the crack propagates to the sides of the loadings shoe instead of straight toward the tips. The load-carrying capacity of PFD was 72 KN, which was less even than the REF, because the surface reinforcement does not overcome the strength lost during installation due to the drilling disturbance caused to the specimen; therefore, the peaks were less compared to the REF wallets.

#### 2.6.3. Steel-Reinforced Specimens

SFS, SCS, and SFD are three different geometrical configurations of steel mesh-reinforced wallets. All these wallets showed minor visible cracks before failure. No total collapse was observed in any of the strengthened steel specimens. The overall shear failure was highly ductile, except for SFD, which showed comparatively less ductile behavior.

#### 2.6.4. Steel Fine Square Mesh Specimens

**Figure 11:**
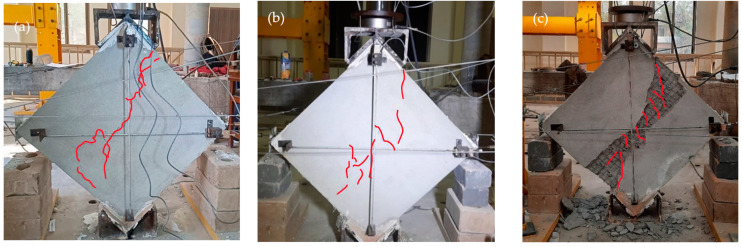
Failure pattern of SFS-reinforced specimens.

The Steel Fine Square (SFS) wallets were reinforced with steel fine square mesh. Figure 11a–c shows the crack pattern of these specimens. They showed a medium spread of shear ductile behavior, as shown in Figure 11c. SFS was able to take a diagonal tension load of 118 KN with a peak stress of 1.22 MPa, corresponding to 0.34% strain. Many microcracks appeared in all directions before the significant crack at the central diagonal. Displacement of the mesh from its original position was observed, ensuring the spread of shear stresses to anchor nodes, although the anchors were not displaced. The mesh changes its perfect square shape with loading. Many mesh wires were broken at the vertical diagonal because the concentrated stresses on wires due to cracks in masonry exceeded the capacity of the wire. SFS wallets took 0.97 MPa at the ultimate point, corresponding to 1.25% strain. No anchor failure was observed. The tests were stopped at 30% degradation. All SFS samples were fully intact and remained able to absorb energy.

#### 2.6.5. Steel Coarse Square Mesh Specimens

**Figure 12:**
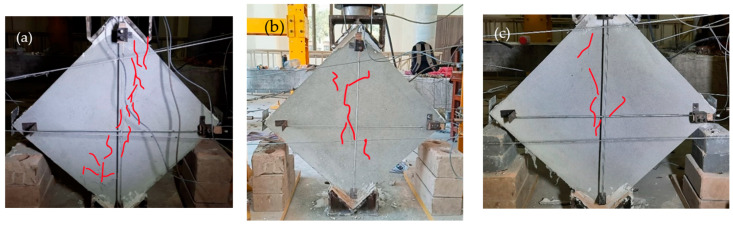
Failure pattern of SCS-reinforced specimens.

The Steel Coarse Square (SCS) specimens were reinforced with steel coarse square meshes. The stress–strain curves include a large horizontal portion with a significant area under the curve, which depicts the high ductility. The SCS specimens were able to take an average peak load of 98 KN and the peak stress was 1 MPa, corresponding to a record-low strain of 0.059%. The crack patterns are shown in Figure 12a–c. Many minor cracks in multiple directions were observed beside the main break. The major cracks initially started at the center in diagonal order and extended to both ends. Following the peaks, the plaster began to fall due to the movement of the meshes, which led to high spread shear failure. The mesh material of SCS is relatively strong and ductile. Therefore, these wallets showed the highest ductility, although their peak strength was relatively low. This could be due to anchor slip, which leads to the repositioning of the mesh. The signs of repositioned mesh were witnessed after plaster removal. The stress at the ultimate point was 0.8 MPa, with a record-high ultimate strain of 1.55%.

#### 2.6.6. Steel Fine Diagonal Mesh Specimens

**Figure 13:**
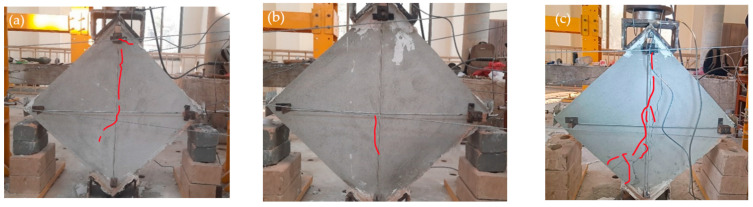
Failure pattern of SFD-reinforced specimens.

The Steel Fine Diagonal (SFD) specimens showed less ductile behavior compared to the SFS and SCS specimens, although they were able to take the highest load of all the wallets at 139 KN. The initial crack patterns are shown in Figure 13a–c. Breakage of the mesh wires was observed at the peak load, and the load capacity dropped suddenly. The peak stress was 1.4 MPa with a strain of 0.346%. Many of the mesh wires were already broken before reaching the ultimate stress point of 1.15 MPa, corresponding to 0.63% strain. The mesh wires are diagonal, and the specimens were tested in a diagonal position; therefore, the wire was parallel to the stress direction. The anchor nodes transfer all the stresses to the mesh, and when the load exceeds the mesh capacity, the wire begins to break. Therefore, these specimens do not show effective post-peak behavior.

## 3. Results and Discussion

### 3.1. Shear Stress and Shear Strain

All shear stress–strain plots in this research are developed as the backbone from the hysteresis of diagonal tension tests. The equations proposed by ASTM E-519 are used to find the shear stress–strain curves. This code interprets it as considering the pure shear at the center of the sample and no axial stresses, with its direction passing through the diagonal ends of specimens [10]. In this case, the shear stresses are principal stresses because the center of the stress Mohr circle lies at the origin of its coordinates. This test method allows us to find the shear stress, strain, and modulus using the provided equations.

(1) $$\sigma_{p}=\tau =0.707\frac{P}{A_{n}}$$

(2) $$A_{n}=\frac{\left(w+h\right)}{2}\;t$$

(3) $$\tau_{m}=0.707\;\frac{P_{m}}{A_{n}}\;$$

(4) $$ϒ=\frac{∆V+∆H}{g}$$

(5) $$G=\frac{\tau }{ϒ}$$

where:

*P_m_* = Diagonal compressive load

*A_n_* = Area

*τ* = Shear stress

*σ_p_* = Principal stresses

*w* = width of the sample

*h* = height of the sample

*t_m_* = Thickness of the sample

*ϒ* = Shear strain

∆*V* = Vertical deformation

∆*H* = Horizontal deformation

**Figure 14:**
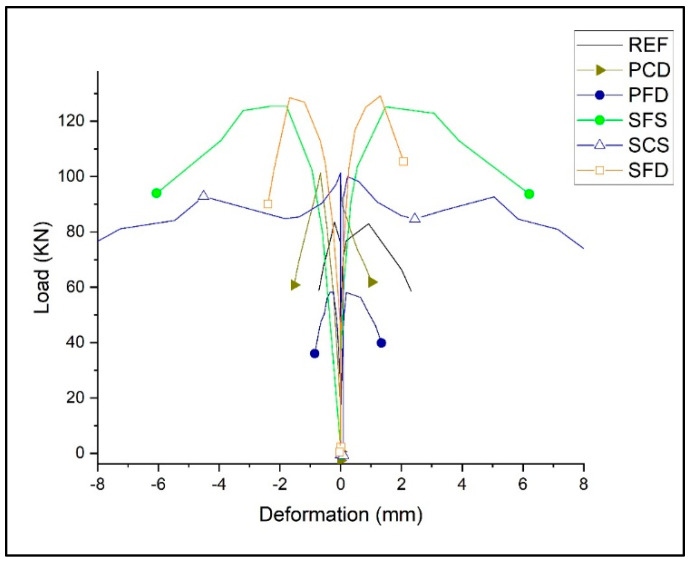
Load–deformation backbone curves of reference and strengthened specimens.

**Figure 15:**
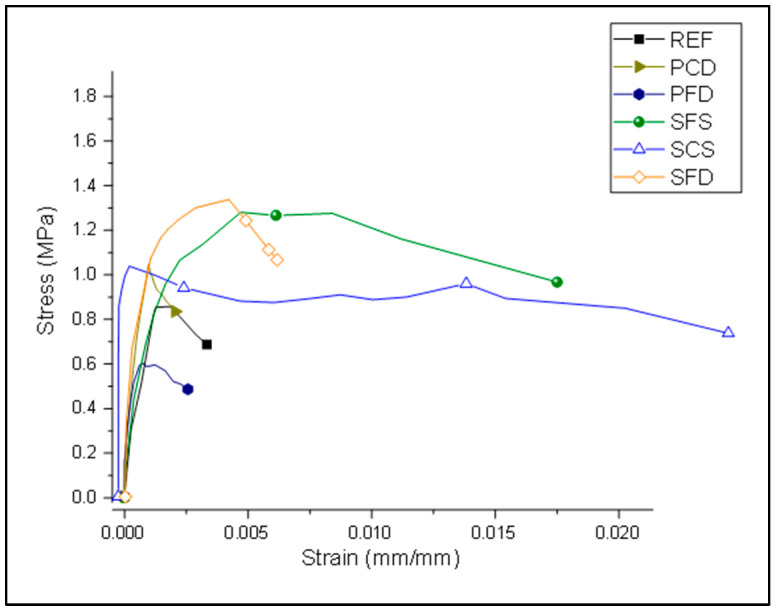
Stress–strain curve of reference and strengthened specimens.

Table 2 shows the average test results obtained from the diagonal tension tests. Figure 14 and Figure 15 show the load–deformation and stress–strain curves of representative specimens. All reinforced specimens showed an increase in shear strength except for the plastic reinforced specimen, PFD, which showed a 17% reduction in load-carrying capacity because the reinforcement could not overcome the strength lost due to the mesh installation process. However, another plastic category (PCD) increased the peak by 18%. A record-high strength increase of 84% was shown by SFD, followed by SFS at a 56% increase compared to the REF specimens. The lowest strength increase among the steel specimens was achieved by SCS, with a strength increase of only 30%. However, it performed very effectively after the yield point.

### 3.2. Ductility

**Figure 16:**
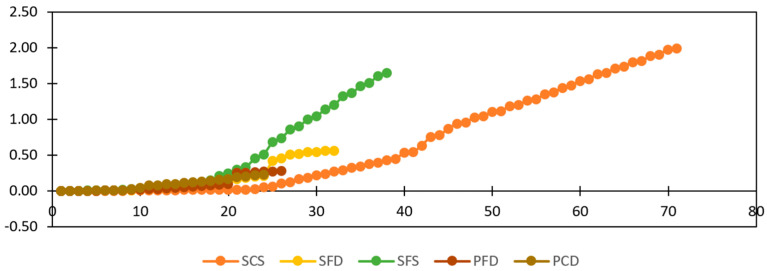
Cumulative energy dissipation.

**Figure 17:**
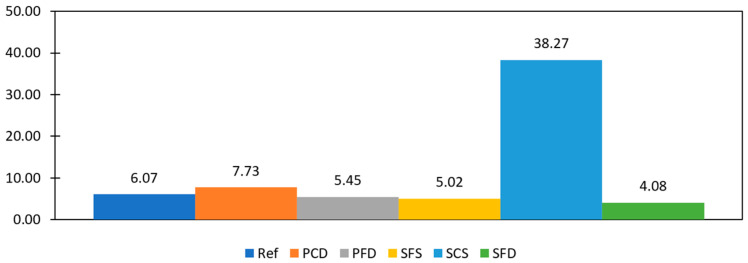
Ductility factor comparison.

Ductility results are presented in Figure 16 and Figure 17. The most crucial factor for the seismic design of brick masonry is ductility. The more ductile the structure, the more energy it can absorb. It measures the deformation ratio from yielding to the ultimate point. Researchers have used many approaches to find the yield point and ultimate point. In this research, a method by Muguruma [35] is used to find the yield and ultimate points on the stress–strain curve:

(6) $$µ=\frac{{∆}_{y}}{{∆}_{u}}$$

where:

∆*_y_* = Displacement at the yield point

∆*_u_* = Displacement at the ultimate point

Ductility is calculated as the strain at the yield point divided by the strain at the ultimate point. As shown in Figure 17, all reinforced specimens showed approximately the same or less ductility compared to the reference specimens, except for one steel category, SCS, which showed six times more ductile behavior. The steel specimens, SFS and SFD, showed high load carrying capacity; however, strains at the yield point were high and strains at the ultimate point were low. Therefore, the calculated ductility is low. On the other hand, SCS showed minimal strain at the yield point and maximum strain at the ultimate point, and therefore achieved the highest ductility.

### 3.3. Energy Dissipation

Energy dissipation is the area enclosed by the load–deformation hysteresis plot. The total energy dissipated by a specimen can be calculated by summing the energy dissipated in all cycles. This is the energy used during friction in the joints and cracks, widening existing cracks, developing new cracks, and crushing bricks and mortar. Figure 16 shows the cumulative dissipated energy of selected specimens from each group. SCS offers the highest energy dissipation at 1.988 KJ, followed by SFS at 1.65 KJ. The high energy dissipation of SCS is due to the spreading of hair cracks, not the brick crushing or the widening prominent cracks. This specimen shows a high spread of ductile failure. SFD showed the least energy dissipation among the steel-reinforced samples because of its sudden strength reduction and low spread of ductile failure. The PFD and PCD specimens dissipated less energy because of their low load-carrying capacity, while The REF specimens dissipated the lowest energy because of their brittle behavior.

### 3.4. Stiffness Degradation

The stiffness degradation (*C_k_*) is the rate of stiffness reduction beyond the yield point. The initial stage is degradation up to the peak point, while the ultimate stage is up to an ultimate point. Stiffness degradation can be measured as the ratio of the secant modulus at a specified point (*K*) to the secant modulus of the yield (*Ky*) point. The Mugurumu [35] elastic perfectly-plastic model is used to find the yield, peak, and ultimate points on the stress–strain curves.

(7) $$C_{K=}\frac{K}{K_{Y}}$$

**Figure 18:**
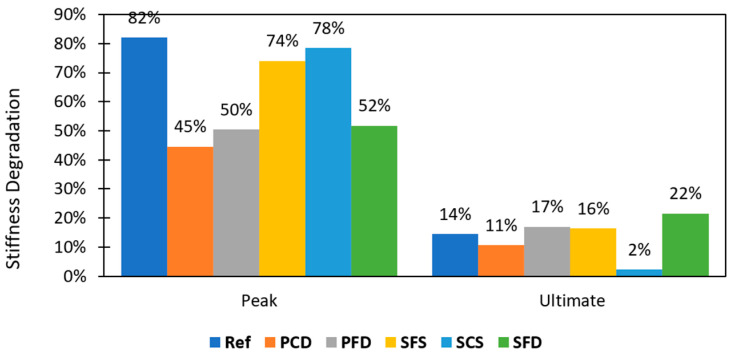
Stiffness degradation.

K_P_ and K_u_ are used to calculate the stiffness degradation and are the slopes of lines passing through the origin, peak (KP), and ultimate point (Ku), respectively. The stiffness degradation was calculated for all the specimens. The average values are shown in Figure 18. The REF showed the highest stiffness degradation at the peak at 82%; SCS was high as well at 78%, followed by SFS and SFD at 74% and 52%, respectively. While SCS had a high value at the peak point, it showed the lowest stiffness degradation at the ultimate stage, at only 2%. This low stiffness degradation of SCS at the ultimate stage shows its significance with respect this parameter.

## 4. Conclusions

The objective of this work is to assess the seismic performance of masonry wallets reinforced with various configurations of steel and plastic meshes. For this purpose, experimental efforts were carried out to determine the seismic strength enhancement of brick masonry using various configurations of steel and plastic meshes as reinforcement. These strengthening systems were compared with unstrengthened samples. Twenty samples were subjected to static cyclic diagonal tension testing. After data analysis, it is concluded that SCS proved to be effective as a seismic strengthening material for brick masonry. It significantly increased the shear strength, energy absorption, and ductility of wallets. These meshes are commercially available, and their installation on wallets is easy and forms a good bond with the masonry with the anchoring technique used here.

The important conclusions of this research work are as follows:

- The diagonal steel mesh wallets (SFD) performed better in terms of shear strength, showing an increase of 84% despite a comparatively lesser quantity of reinforcement. Because the mesh wires were parallel to the direction of the stresses, the mesh wires behaved as tension and compression struts. Moreover, SFS and SCS showed an increase of 56% and 30%, respectively.
- The SCS category proved to be very effective for increasing energy dissipation; the capacity increased to 1.988 KJ, which is fourteen times that of the REF specimens. Likewise, SFS increased the energy dissipation eleven-fold. This significant energy dissipation is due to the spread of hairline cracks in the masonry and minor deformation in the meshes, although no anchors failed.
- SCS performed the best in terms of ductility, achieving a six-fold increase compared to the REF specimens. All remaining specimens (PFD, PCD, SFS, and SFD) did not show any improvement in ductility.
- Stiffness degradation occurs due to reversal load. All specimens degraded at the initial stage (52% to 82%). The degradation at the initial stage was highest for SCS (after the REF specimens), however, it was only 2% at the ultimate stage. The remaining reinforced specimens (PCD, PFD, SFS, and SFD) fell between 11% and 22% degradation.
- Failure modes play an essential role in the seismic performance of brick masonry. The high spread ductile failure mode dissipated more energy along with ductile behavior, as observed in the SCS specimens, as opposed to the SFD specimens, which had a lower ductility factor and low energy dissipation despite their high load-carrying capacity.
- SCS is recommended as a reinforcement material because it performed very effectively in terms of the important seismic parameters. While the shear strength improved by only 30% (from 75.4 to 98.1 KN), energy dissipation improved fourteen-fold, from 0.138 to 1.988 KJ. Furthermore, the ductility improved six-fold, from a factor of 6 to 38. Furthermore, the stiffness degradation of SCS was only 2% at the ultimate stage.
- Considering the significant performance of SCS-reinforced material, it represents an effective option for strengthening vulnerable brick masonry structures in high-risk seismic zones.

## Figures and Tables

**Table 1:** Summary of the strengthening scheme.

Sample Code	No. of Specimens	Strengthening Mesh Material	Wires Spacing (mm)	Diameter (mm)	Yield Strength (MPa)	Ultimate Strain (%)	% Volume Reinforcement
REF	5	Un-reinforced	-	-	-	-	-
PCD	3	Plastic Coarse Diagonal	16.09	1.76	27	600	0.13
PFD	3	Plastic Fine Diagonal	13.75	1.77	27	600	0.15
SFS	3	Steel Fine square	26.34	1.68	866.4	0.63	0.073
SCS	3	Steel coarse square	57.5	2.5	1000.1	2.1	0.074
SFD	3	Steel fine Diagonal	27.16	0.88	870.7	1.8	0.034

**Table 2:** Diagonal static cyclic compression test results.

Specimen	Peak Load (KN)	Shear Strain at Yield Point%	Shear Stress at Yield Point (Mpa)	Shear Modulus at Yield Point (Mpa)	Shear Strain at Peak %	Shear Stress at Peak (Mpa)	Shear Modulus at Peak Point (Mpa)	Shear Strain at Ultimate %	Shear Stress at Ultimate (Mpa)	Shear Modulus at Ultimate (Mpa)	Ductility Ratio	Energy AbsorptionKJ
REF	75.4	0.06	0.75	1778.4	0.087	0.78	1212.8	0.36	0.36	171.9	6.07	0.138
PCD	89.0	0.06	0.88	1630.8	0.147	0.92	739.1	0.48	0.48	154.0	7.73	0.278
PFD	62.5	0.04	0.58	1546.3	0.093	0.64	717.9	0.22	0.22	238.9	5.45	0.223
SFS	118.2	0.25	1.14	529.5	0.348	1.22	371.0	1.25	1.25	78.0	5.02	1.65
SCS	98.1	0.04	1.0	3814.1	0.059	1.03	3138.7	1.55	1.55	51.4	38.27	1.988
SFD	139.0	0.15	1.29	858.3	0.346	1.43	427.4	0.63	0.63	181.3	4.08	0.563

## Data Availability

All the data used in the study has been reported in the manuscript. For any ambiguity, the corresponding authors may be contacted.
